# Patient and Health Care Provider Perspectives on Patient Access to Test Results via Web Portals: Scoping Review

**DOI:** 10.2196/43765

**Published:** 2023-10-19

**Authors:** Olga Petrovskaya, Albina Karpman, Joanna Schilling, Simran Singh, Larissa Wegren, Vera Caine, Elizabeth Kusi-Appiah, Willow Geen

**Affiliations:** 1 School of Nursing University of Victoria Victoria, BC Canada; 2 Faculty of Nursing MacEwan University Edmonton, AB Canada; 3 Faculty of Nursing University of Alberta Edmonton, AB Canada; 4 South Health Campus, Women's Health Calgary, AB Canada

**Keywords:** patient portal, web portal, MyChart, electronic health records, personal health records, patient access to records, laboratory tests, radiology reports, diagnostic imaging, laboratory test results, result release, embargo, the Cures Act

## Abstract

**Background:**

A frequently used feature of electronic patient portals is the viewing of test results. Research on patient portals is abundant and offers evidence to help portal implementers make policy and practice decisions. In contrast, no comparable comprehensive summary of research addresses the direct release of and patient access to test results.

**Objective:**

This scoping review aims to analyze and synthesize published research focused on patient and health care provider perspectives on the direct release of laboratory, imaging, and radiology results to patients via web portals.

**Methods:**

PRISMA (Preferred Reporting Items for Systematic Reviews and Meta-Analyses) guidelines were followed. Searches were conducted in CINAHL, MEDLINE, and other databases. Citations were screened in Covidence using the inclusion and exclusion criteria. Primary studies that focused on patient and health care provider perspectives on patient access to laboratory and imaging results via web portals were included. An updated search was conducted up to August 2023. Our review included 27 articles—20 examining patient views, 3 examining provider views, and 4 examining both patient and provider views. Data extraction and inductive data analysis were informed by sensitizing concepts from sociomaterial perspectives, and 15 themes were generated.

**Results:**

Patient perspectives (24 papers) were synthesized using nine themes: (1) patterns of use and patient characteristics; (2) emotional response when viewing the results and uncertainty about their implications; (3) understanding test results; (4) preferences for mode and timing of result release; (5). information seeking and patients’ actions motivated by viewing results via a portal; (6) contemplating changes in behavior and managing own health; (7) benefits of accessing test results via a portal; (8) limitations of accessing test results via a portal; and (9) suggestions for portal improvement. Health care provider perspectives (7 papers) were synthetized into six themes: (1) providers’ view of benefits of patient access to results via the portal; (2) effects on health care provider workload; (3) concerns about patient anxiety; (4) timing of result release into the patient portal; (5) the method of result release into the patient portal: manual versus automatic release; and (6) the effects of hospital health information technology system on patient quality outcomes.

**Conclusions:**

The timing of the release of test results emerged as a particularly important topic. In some countries, the policy context may motivate immediate release of most tests directly into patient portals. However, our findings aim to make policy makers, health administrators, and other stakeholders aware of factors to consider when making decisions about the timing of result release. This review is sensitive to the characteristics of patient populations and portal technology and can inform result release framework policies. The findings are timely, as patient portals have become more common internationally.

## Introduction

### What Is a Patient Portal?

Health care stakeholders have been increasingly encouraged to respect and promote the active role of patients in shared decision-making and care. This shift is supported by consumers’ quick and convenient access to health information, including personal health information, via information and communication technology (ICT) such as patient portals, the internet, and social media. Patient portals are web-based platforms that provide patients with access to their personal health information contained in the health organization’s electronic health record (EHR). In other words, patient portals are tethered to an EHR. By creating a portal account, patients are provided with secure and convenient access to their information, which can facilitate their active engagement in their care. Patient portals can provide access to diagnosis, laboratory and diagnostic imaging results, medication lists, booking and viewing appointment times, sending and receiving secure messages with their health care team, and in some cases requesting prescription refills and conducting video visits, among other functionalities. Although the above definition and list of functionalities of patient portals are commonly used in the literature [1,2], taxonomies of patient portals offer a more systematic way of describing this technology [3].

One example is the recent Taxonomy of Patient Portals based on Characteristics of Patient Engagement [3] developed for health information managers and updated based on a Delphi study with 13 participants (mostly in senior roles as health informatics specialists), with experience in patient portals ranging from little to significant, from Austria, Germany, and Switzerland. The Taxonomy of Patient Portals based on Characteristics of Patient Engagement describes the patient portal in terms of 7 aspects that cover 25 dimensions with 65 characteristics. The key criterion underlying this taxonomy is the level of patient engagement. The aspects (and examples of dimensions) are portal design (eg, care sector target, medical specialty, or patient target such as outpatient), management (eg, appointment booking and prescription renewal), communication (eg, e-consult), instruction (eg, patient education), self-management (eg, visit preparation), self-determination (eg, declaration of will and study sign-up), and data management (eg, record access, health data amend, or upload) [3].

### Background

A large body of research including primary studies [4-11] and reviews [1,2,12-14] aimed to evaluate the effects of patient access to EHR. A systematic review of 10 randomized controlled trials concluded that the effects of portals are uncertain when compared with usual care [1]. Other reviews encompassed primary studies with various designs, including qualitative and nonexperimental designs, and their findings were diverse.

Brands et al [12] reported high levels of patient satisfaction and acceptability of portals. Portal use improved patients’ understanding of their health conditions [12], monitoring of health status, patient-physician interaction, and quality of care [13]. Portals promoted the use of recommended care services [12], but the results were mixed for portal effect on reducing physician and emergency department (ED) visits [13]. Contrary to what might have been anticipated by portal advocates, patients with comorbidities and a high disease burden seemed to benefit less from portals [12].

Although the beneficial effects of portals are more noticeable when patients use the portal’s active features [12] (eg, communication with health care teams, systematic monitoring of laboratory values to adjust lifestyle, and information upload), most commonly, portals are used passively to access information [14]. Patients avoid generating and managing their health data in the portal because of concerns about data validity, applicability, and confidentiality [14].

In a recent study reporting health care provider (HCP) perspective from 673 general practices in the Netherlands, 42% described their experiences with patients’ web-based access to medical records as neutral and 37% as mostly positive [4]. Two-thirds reported an increase in e-consultations and administrative work [4]. Patients’ perceptions of a Finnish portal [5] and Norwegian portal [10] were positive, with managing prescriptions and viewing test results and medical notes being the most useful [5]. Portals facilitate communication with health care teams and the monitoring of health status and care activities [7,8,10] by motivating patients to ask questions, prepare for medical appointments, and share documents with other providers [8,10]. Liu et al [6] found an indirect relationship between portal use and cancer survivors’ psychological and physical health, mediated by patient-centered communication and self-efficacy. The analysis of a large data set from primary care in the United States found overall inconsistent effects of portal use, but patients who used messaging and viewed laboratory results more often exhibited a larger reduction in no-shows compared with other user subgroups [11]. However, not all patients want to use portals and may consider them unnecessary, impersonal, incomprehensible, misery oriented, fear provoking, energy demanding, cumbersome, and impoverishing (ie, negatively changing individual and social life) [9].

One of the portal features that patients use the most is viewing their laboratory and diagnostic imaging test results [15,16]. Analyses of a system’s data such as portal logs in large medical centers in the United States [15] and Canada [16] demonstrated that viewing test results was the second most used portal functionality, whereas the users of the Swedish national portal identified this feature as the most important [17]. Currently, the number of research publications reporting why and how patients access their laboratory and diagnostic tests via web-based portals and what the implications of this access are for patients, HCPs, and health systems is increasing. The COVID-19 pandemic has added urgency to the use of technology to support the online delivery of health care services. However, to date, the literature has been dispersed, and no overall synthesis has been reported. Part of our interest in undertaking this review is explicitly regarding the timing of the release of test results to patients. The results can be manually released by HCPs or autoreleased either immediately or after a predetermined delay. In discussions surrounding the embargo period, increased patient anxiety and harm to patients are often cited as concerns by opponents of immediate result release. However, American health care organizations might be opting for a contentious immediate result release after the implementation of the 21st Century Cures Act.

The 21st Century Cures Act, enacted in the United States in 2016, encourages patients’ unrestrained access to electronic health information and promotes interoperability among EHR vendors [18]. The Cures Act Information Blocking Provision required the implementation of patient access by April 2021, with significant consequences for noncompliant health organizations and HCPs [18]. Importantly, this rule “does not increase the type of health information that patients and families can access; it only facilitates automatic release via patient portals and easier access electronically” [18]. In particular, the Cures Act has direct implications for the release of test results into patient portals. Although the Act did not require that all tests be released automatically, but rather upon a patient’s request, some health systems weighed the logistics and chose to revise their result release frameworks to eliminate embargo periods for most tests [19]. In other words, some health systems in the United States switched to the immediate release of nearly all laboratory and imaging results, including those considered sensitive and suitable exclusively for in-person discussions [19]. This policy context in the US foregrounds the importance of understanding past and present practices and experiences of patient web access to their test results.

### Objectives

The purpose of this scoping review was to analyze and synthesize published research focused on patient and HCP perspectives on patient web-based access to their laboratory and imaging tests. This review was guided by the following research questions: What are the experiences and perceived advantages and limitations for patients and family caregivers who access their laboratory and diagnostic test results via web portals? What do HCPs perceive as the benefits and drawbacks of direct patient access to test results? What factors should be considered when implementing patient access to test results via portals? And What is known specifically about the timing of result release and the effects of timing in relation to other important considerations for patients and HCPs?

## Methods

### Overview

**Figure 1:**
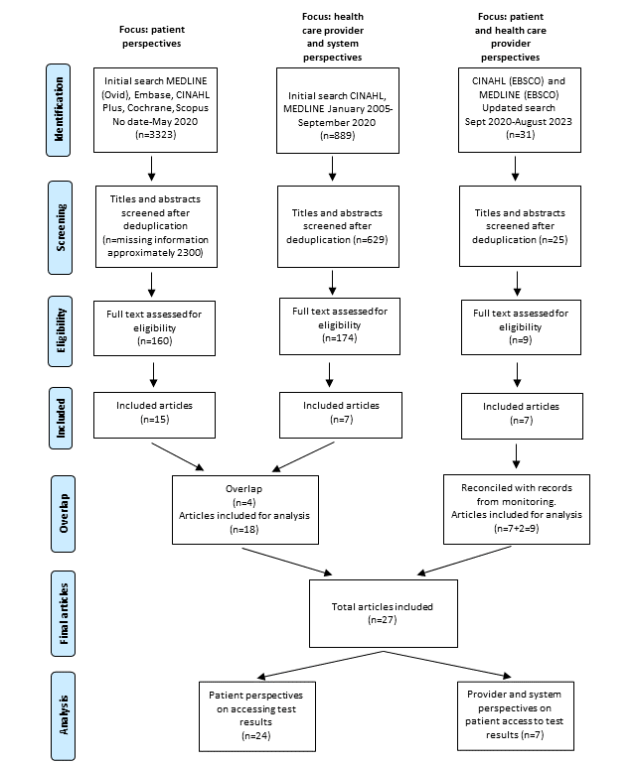
PRISMA flowchart.

This review followed a modified scoping review methodology previously used by the first author in published scoping reviews [20], which is based on selected recommendations from the Joanna Briggs Institute for scoping reviews [21] in combination with the PRISMA-ScR (Preferred Reporting Items for Systematic Reviews and Meta-Analyses extension for Scoping Reviews) [22]. Initially, part of this review was undertaken as a graduate student project, which explained the multiple timelines of the searches. The review process is depicted in the PRISMA (Preferred Reporting Items for Systematic Reviews and Meta-Analyses) flowchart (Figure 1).

### Data Sources and Search Strategy

Web-based literature searches focused on patient access to laboratory or imaging results via patient portals. The initial searches were conducted at 2 points in 2020, in consultation with an academic librarian. In May 2020, MEDLINE (Ovid), Embase, CINAHL Plus, Cochrane, and Scopus were searched using both subject heading and keyword terms for articles available in English without time restrictions. To increase specificity and precision, in September 2020, the keyword search within titles and abstracts in CINAHL and MEDLINE was limited to academic journals published in English from 2005 to 2020. The results were exported to the Covidence software (Veritas Health Innovation Ltd) for screening. From September 2020 to August 2023, OP monitored new publications in key health informatics journals. In addition, an updated search was conducted in August 2023 using keyword searches within titles and abstracts in CINAHL (EBSCO) and MEDLINE (EBSCO) from September 2020 to August 2023. Multimedia Appendix 1 provides the complete search strategies.

### Eligibility Criteria

Articles were selected based on the following inclusion criteria: (1) phenomenon of interest: patients accessing their laboratory or diagnostic imaging test results via patient portals; (2) population: patients or family caregivers, HCPs, and health systems; (3) technology: tethered patient portals, personal health records that allow patient access to laboratory or imaging results, or web portals for patients accessing laboratory and imaging results (we were interested in actual, existing portals or usability testing of actual portal prototypes designed for clinical settings); and (4) the type of publications: primary peer-reviewed research of any design (qualitative, quantitative, and mixed methods), *directly* focused on the phenomenon of interest.

We excluded studies that (1) tangentially mentioned the percentage of patients viewing test results or mentioned patients hypothetically interested in viewing test results; (2) addressed other forms of ICT and patient access to information other than test results; (3) focused on genetic portals, which are contextually unique in that genetic tests are not routinely released into mainstream patient portals but rather specialized portals are designed (one example is the study by Williams et al [23]); (4) focused on inpatient portals for hospitalized patients, which are contextually unique areas requiring separate analyses; (5) focused on software development and implementation as well as portals developed for research purposes (one example is the study by Fraccaro et al [24]); (6) used hypothetical scenarios on simulated patients (one example is the study by Bar-Lev and Beimel [25]); and (7) were situated outside of high-resource countries (this was seen as contextually unique). We also excluded review papers and gray literature. This review was limited to primary research, and the analysis of other sources was outside the scope of this study. However, our findings suggest the potential usefulness of analyzing selected gray sources, such as health care organizations’ result release portal policies. Refer to Multimedia Appendix 1 for detailed tables of the inclusion and exclusion criteria.

### Study Selection

We used the Covidence systematic review management software to remove duplicates and screen citations. Refer to the PRISMA flowchart for details (Figure 1). In May 2020, LW screened titles and abstracts, followed by LW and OP’s independent review of the selected full texts, and disagreements were resolved by consensus. During this stage, 15 articles *addressing patient perspectives* met the inclusion criteria [26-40]. In September 2020, SS and OP independently screened titles and abstracts, followed by an independent review of the selected full texts, and disagreements were resolved by consensus. During this stage, 7 articles *addressing HCP perspectives* met the eligibility criteria, of which 3 were new [41-43], whereas 4 were already identified in the previous search as they included both patient and HCP perspectives [29,35,36,39]. In the updated search in August 2023, OP and WG independently screened titles and abstracts, followed by an independent review of the selected full texts, and disagreements were resolved by consensus. During this stage, 7 articles met the inclusion criteria [19,44-49]. A list of articles compiled by OP during the ongoing monitoring of new publications from September 2020 to August 2023 was compared with the results of the updated search, and 2 new articles [50,51] meeting the inclusion criteria were added.

In total, 27 unique articles were included in the data extraction, analysis, and synthesis, 4 of which captured the perspectives of both patients and HCPs. The articles focused on patient perspectives (n=24) and HCP perspectives (n=7) were thematically analyzed, and the results were reported separately for each group. This granular approach produced a comprehensive summary sensitive to patient characteristics and portal technology, which is a step toward building evidence to inform the result release framework policies and help HCPs appreciate the benefits and challenges that patients report when viewing their test results via web portals.

### Data Extraction, Analysis, and Synthesis

LW, SS, and OP developed and piloted the Microsoft Excel table for data extraction. Categories for data extraction included citation, country, health care setting, portal type or brand, study purpose, design, methods of data collection, sample, findings (separately from patients and providers and for each method of data collection, eg, self-reports vs system log analysis), and limitations. At different stages of the review project, LW, SS, AK, JS, and WG extracted data from the included articles, whereas OP, EKA, AK, and JS reviewed the extracted data for completeness and accuracy.

Data extraction and thematic analysis were informed by sensitizing concepts and insights from sociological practice theory and science and technology studies. Specifically, sociomateriality and actor-network perspectives summarized elsewhere by the first author [52-54] and translational mobilization theory [55] describing the invisible organizing work of HCPs (ie, nurses) provided valuable lenses for our engagement with the articles. Thus, theme generation has been informed by assumptions about the benefits of contextualized descriptions, technology as agential, the importance of viewing patient portals as *interacting* in patients’ everyday lives that involve multiple human and nonhuman elements, and health care practices as encompassing not only direct caregiving roles but equally important the organizing work. This organizing work includes the creation of working knowledge, patient care trajectory articulation, and transitions of care [55], all of which change with the introduction of EHR and patient portals.

At the text level, thematic analysis involved a hermeneutic process of understanding the whole to understand each part and vice versa. This process was necessary to contextualize the findings reported in each article. We looked for content and patterns present across the findings of the reviewed studies to propose and populate initial themes (eg, negative emotions patients feel when viewing test results) and for findings mentioned infrequently (eg, patients’ comments about the timing of result release). Themes were generated inductively and were not limited to the formal themes (subheadings) proposed in the original studies. Examples include the rarely articulated or novel themes related to the test result-release framework (mode and timing), patients’ views of portals as archives, patients serving as a link connecting fragmented health services because of patients’ ability to share test results with providers lacking EHR access, and a more nuanced presentation of factors contributing to patient anxiety when viewing their test results via portals.

## Results

### Overview

The 27 reviewed articles were published between 2007 and 2023, with the majority (n=22, 81%) published in 2016 or later. Studies were conducted in the United States (n=21, 78%), the Netherlands (n=3, 11%), Canada (n=2, 7%), and Denmark (n=1, 4%) using quantitative (n=17, 63%), mixed methods (n=9, 33%), and qualitative designs (n=1, 4%). The most frequently used methods of data collection were surveys (n=16, 59%), interviews (n=8, 30%), and analyses of EHR data (n=9, 33%). Where surveys or interviews were used, the samples consisted of patients, family caregivers, and HCPs, such as physicians, oncologists, or nurses. Among 17 studies that provided the portal name or brand, at least 8 (47%) studies were conducted in health care facilities using the EPIC patient portals. Three studies [19,27,31] were conducted in the same setting, University of Iowa Hospitals and Clinics, and involved analyses of the EPIC Reporting Workbench from 2016, 2017, and 2020 to 2021.

Most reviewed papers reported patient access to a variety of test results, mostly laboratory but also imaging. A subgroup of articles originating in the United States focused specifically on patient access to radiology and imaging results [26,29,30,33,34,40,51]. This focus appears to reflect a unique practice in this country whereby (1) radiology services often implement their own web portals (rather than, or in addition to, supplying results to the patient portals tethered to the main EHRs) and (2) radiologists can directly interpret the results to patients. According to Mangano et al [33], the Radiological Society of North America tried to encourage radiologists to enhance their visibility among patients, including the practice of direct communication of imaging results to patients, which might be one factor explaining the relatively high number of studies focused on releasing imaging results. We report findings from these articles from either patient perspectives or HCP perspectives depending on the sources of data.

The detailed participant groups, sample sizes, health care settings, and portal technology are presented in Table 1.

Table 2 lists 9 themes generated from 24 studies focused on patient perspectives and 6 themes generated from 7 studies focused on HCP perspectives. If the study included data from patients and HCPs [29,35,36,39], we reported these findings separately, in respective sections. Williams et al [42] does not address patient access to tests results but provides important findings on the effects of physician access to electronic test results. For this reason, we decided to report the findings of this study. Krasowski et al [31] study was difficult to categorize as it mostly supported themes in the patient perspective section while also providing valuable data about health organization’s result release practices. We extracted all these data but placed this study [31] in the patient perspective group.

**Table 1:** Study characteristics.

Author and country	Study objective	Research design; method of data collection	Participants and sample size	Participant demographics and health condition	Health care, setting, and portal technology
Baun et al [50], Denmark	Explore experiences of women with cancer using EHR^a^ to view imaging results	Mixed methods; survey and interviews	Patients (women undergoing scans every 3 mo); 38 surveyed and 4 interviewed	Survey respondents: White, aged 42-84 (median 69) y; interview informants: aged >40 y; 23 portal users	Department of Nuclear Medicine, Odense University Hospital; Denmark’s national portal
Edmonds et al [26], United States	Identify patient characteristics associated with use of portals to view their bone density results	Quantitative nonexperimental; survey	649 patients viewing bone density scan via portal	Aged >50 (mean 64) y; other data reported for a larger sample, including portal nonusers	Two sites: UI^b^ and KPGA^c^; EPIC portals: MyChart (UI) MyHealthManager (KPGA)
Foster and Krasowski [27], United States	Examine portal activation and access to diagnostic tests by ED^d^ patients	Quantitative nonexperimental; retrospective analysis of EPIC Reporting Workbench for 12 mo in 2016-2017	Patients with at least 1 ED encounter and 1 test; data sets: 208,635 laboratory tests on 25,361 unique patients; 23,504 radiology studies on 14,455 unique patients. Approximately 37% of patients had a portal account	All ages	Emergency department; UI hospitals and clinics (same as [19,31]); EPIC portal
Giardina et al [28], United States	Explore patients’ experiences with accessing test results via portals	Mixed methods; descriptive statistics and interviews	95 patients	Average age 54.6 y; 56% male; 65% White; 62% with one or more chronic conditions; 72% use portal for at least 1 y	4 large outpatient clinics in Houston, including primary care clinics and VA^e^ facilities; MyChart (EPIC) and MyHealth*e*Vet
Henshaw et al [29], United States	Examine patients’ and referring HCPs’^f^ experiences of manually releasing radiology reports (no images)	Mixed methods; descriptive statistics, patient survey, HCP survey, and group interview	508 patients; 48 referring HCP (physicians, physician assistants, and nurse practitioners)	Not reported	Kaiser Permanente Hawaii, primary care and specialty clinics; Kaiser Permanente portal
Hiremath et al [30], United States	Examine patient perceptions of a pilot access to images and radiology reports	Quantitative nonexperimental; survey	456 patients	Aged 18-86 (mean 52) y; 64% female; over 80% used computer at least daily	Outpatient-imaging center; Image Share Project by the Radiological Society of North America
Hulter et al [44], Netherlands	Explore patient preferences for timing of result release	Mixed methods; portal use data and interviews	4592 patients who indicated in the portal their preference for timing of tests; 7 patients interviewed	36% male, mean age 56 (SD 15) y; 64% female, mean age 50 (SD 16) y	Dutch teaching hospital; portal brand not reported
Krasowski et al [31], United States	Evaluate variations in results release (automated vs manual) and subsequent patient access to the portal	Quantitative nonexperimental; retrospective analysis of EPIC Reporting Workbench for 6 mo in 2016	Approximately 1.6 million results (anatomic pathology, lab, and radiology) for nearly 60,000 unique patients; anecdotal accounts	All ages	Outpatient, inpatient, emergency departments; UI hospitals and clinics; EPIC portal (same as [19,27])
Mák et al [32], Canada	Explore patient comprehension and anxiety when viewing laboratory test results	Quantitative nonexperimental retrospective cohort study, survey, and comparison of portal users with nonusers	2047 patients with portal access and at least 1 test in last 12 mo	Age: 62% above 55 y; 62% female; 77% rated health as excellent or good; 60% had chronic condition; 62% had at least 3 tests in last 12 mo	Preexisting laboratory database; dedicated portal to access laboratory results in British Columbia, Canada
Mangano et al [33], United States	Survey patients about their preferred method of receiving radiologic results and whether radiologists should communicate results directly to patients	Quantitative nonexperimental; survey	642 patients undergoing contrast-enhanced CT^g^ or MRI^h^	Age 18-80+ (mode 51-60) y; 87% had internet access; 44% aware of web access to radiology reports; 47% of those viewed imaging results	Large academic tertiary care medical center that operates 2 outpatient-imaging facilities; portal (unspecified) allows access to all kinds of test results and doctors’ notes
McFarland et al [45], United States	Examine portal enrollment, use and rates of patients viewing radiology and laboratory results	Quantitative; nonexperimental; analysis of EHR data	424,422 patient records; 138,783 portal users	Mean age 49 (SD 21) y; 58% female; 58% White, 30% Black; 33% enrolled in patient portal	Single academic tertiary care center; Oracle Cerner portal
Miles et al [34], United States	Evaluate frequency of viewing radiology reports and demographic factors associated with report viewing	Quantitative nonexperimental analysis of system logs	61,131 patients with at least 1 radiology report	18-80+ y	University of Washington, medical center; University of Washington eCare portal
Norris et al [51], United States	Examine experiences and actions of patients accessing radiology results	Mixed methods; survey (closed and open-ended questions)	299 patients	58.5% aged 55+ y; 69% female	UCHealth^i^; MHC^j^ portal
Okawa et al [35], United States	Compare physician patterns of releasing reports manually vs autorelease and examine patient viewing patterns	Quantitative nonexperimental; analysis of system data–number of reports released into portal and viewed by patients	Total number of reports available to patients in the portal 86,659 in 2015	Reports released for 52,293 unique patients in 2015, of whom 56% were active on the portal	Outpatient-imaging center, outpatient departments, and EDs; Kaiser Permanente, Hawaii; Kaiser Permanente portal
Pillemer et al [36], United States	Examine impact of allowing patients to view their test results via patient portal	Mixed methods; interview with patients and physicians, survey of patients who are portal users, analysis of EHR data (service use pre- and postdirect result release; test viewers vs nonviewers), and portal use data	6368 patients completed survey; 13 patients with HbA_1c_^k^ or abnormal Papanicolaou result interviewed; sample size for HCP not specified; portal use and EHR data: 77,901 results released to 14,441 patients of whom test viewers, n=8486	Patients test-viewers: mean age 51 y; 54% male; 91% White	UPMC^l^ outpatient practices; EPIC MyChart branded as MyUPMC
Robinson et al [37], Canada	Understand why patients access laboratory results and impact on their health	Qualitative; interviews	21 patients	Age: 18-80+ y; 62% between 60 and 79 y; 57% male; healthy to chronic illness	Primary Care Centre; EpicCare and myCARE portals
Rodriguez et al [41], United States	Compare views of oncology nurses and physicians on patient access to laboratory results pre- and postimplementation and impact on workload	Quantitative nonexperimental; survey and nursing workload (number of phone calls received from patients regarding laboratory results)	HCP: 187-251 nurses surveyed, 10 of them participated in workload study; 66-100 attending physicians surveyed	Nurses: mostly female, aged 25-54 y; physicians: 60% male, aged 35-54 y; majority confident in computer skills	Outpatient department of Memorial Sloan-Kettering Cancer Center, New York; Portal MyMSKCC, vendor not reported.
Schultz and Alderfer [38], United States	Explore caregivers’ preferred method of receiving test results and the disadvantages of portals	Mixed methods; interviews and survey	19 family caregivers of children with cancer	Parents aged 25-49 (mean 40) y; 79% female; 26% Black or African American; pediatric oncology	Oncology clinic; EPIC MyChart (MyNemours)
Talboom-Kamp et al [46], Netherlands	Investigate experiences and self-efficacy of patients using portal to view laboratory results	Quantitative nonexperimental; survey (eHealth impact questionnaire)	354 patients who are portal users	Not reported	Saltro, a primary care diagnostic center and laboratory; Saltro patient portal for laboratory results
Tossaint-Schoenmakers et al [47], Netherlands	Examine effect of patient characteristics on usability and self-efficacy when accessing laboratory results	Quantitative nonexperimental; survey	748 patients	Mean age 58.5 y; 57% female; 57% highly educated (bachelor’s or higher); 68% reported no chronic illness	Diagnostic center; dedicated laboratory portal
Wakefield et al [48], United States	Examine the association between portal use and care coordination between multiple HCP through comparing duplication of HbA_1c_	Quantitative nonexperimental; portal use data, Medicare records, and comparison between portal users and nonusers	30,186 veterans who use both VA and non-VA health services	Age: 25-85+ y; 98% male; 90% White; all patients with diabetes	VA and Medicare health facilities; MyHealth*e*Vet Portal
Wald et al [39], United States	Feasibility pilot of patient access to their laboratory results to understand technical, workflow, and organizational challenges	Quantitative nonexperimental; survey and spontaneous comments	128 patients surveyed 12 wk after pilot began; 10 physicians provided spontaneous and solicited feedback 8 wk after pilot began	Patients: mean age 42 y; 49% female	Two primary care practices; Eastern Massachusetts; Patient Gateway
Williams et al [42], United States	Analyze influence of organizational and technology characteristics on patient quality outcomes	Quantitative nonexperimental	System data from 1039 American hospitals; 2 databases (health information and management systems society analytics survey+center for Medicare and Medicaid service)	N/A^m^	Hospitals
Winget et al [43], United States	Examine perspectives of oncologists about autorelease of pathology and radiology reports after 7-d embargo	Mixed methods; survey: descriptive statistics and thematic analysis of comments	82 oncologists completed survey, 35 of whom provided comments	Not reported	Stanford Cancer Center; portal brand not reported
Wood et al [19], United States	Examine changes in patient reviewing patterns before and after switch to immediate release of nearly all laboratory and imaging results	Quantitative nonexperimental; retrospective pre (10 mo)-post (10 mo) study and analysis of data from EPIC Reporting Workbench in 2020-2021	3,809,397 diagnostic tests from 204,605 unique patients; 56.5% female; 84% White; 96.5% preferred English as their primary language; 71% with active portal account	All ages	ED, inpatient, outpatient; departments; UI hospitals and clinics; EPIC MyChart (same as [27,31])
Woolen et al [40], United States	Determine timing of imaging result release based on patients’ experience of portal use	Quantitative nonexperimental; survey	418 patients with cancer, 43% of whom had at least some experience of portal use	Aged 11-65+ y; majority 50-64 y; 60% female; 66% White; 26% with cancer, depression, and cardiovascular disease	4 outpatient sites from 2 institutions in 2 Midwestern states; portal brand not reported
Zhang et al [49], United States	Examine patients experience with comprehending laboratory results	Mixed methods; interviews and survey	203 patients surveyed; 13 patients interviewed	Survey: aged 18-80+ y; most aged 26-49 y; 51% male, 69% White; interview: aged 18-64 y; 46% aged 26-49 y; 76% White; 70% female; 85% technology proficient	Health setting not reported; portal brand not reported

^a^EHR: electronic health record.

^b^UI: University of Iowa.

^c^KPGA: Kaiser Permanente of Georgia.

^d^ED: emergency department.

^e^VA: Veteran Affairs.

^f^HCP: health care provider.

^g^CT: computed tomography.

^h^MRI: magnetic resonance imaging.

^i^UCHealth: University of Colorado Health.

^j^MCH: My Health Connection.

^k^HBA_1c_: glycated hemoglobin.

^l^UPMC: University of Pittsburgh Medical Center.

^m^N/A: not applicable.

**Table 2:** Themes and articles supporting each theme (n=27 studies).

Themes		Studies
**Patient perspectives (n=24)**		
	Patterns of use and patient characteristics	Baun et al [50], Edmonds et al [26], Foster and Krasowski [27], Henshaw et al [29], Hiremath et al [30], Krasowski et al [31], Mangano et al [33], McFarland et al [45], Miles et al [34], Norris et al [51], Pillemer et al [36], Robinson et al [37], Wald et al [39], Wood et al [19], Woolen et al [40]
	Emotional response when viewing the results and uncertainty about their implications	Baun et al [50], Giardina et al [28], Krasowski et al [31], Mák et al [32], Norris et al [51], Pillemer et al [36], Robinson et al [37], Schultz and Alderfer [38], Zhang et al [49]
	Understanding test results	Baun et al [50], Giardina et al [28], Hulter et al [44], Mák et al [32], Norris et al [51], Robinson et al [37], Schultz and Alderfer [38], Zhang et al [49]
	Preferences for mode and timing of result release	Baun et al [50], Giardina et al [28], Hulter et al [44], Pillemer et al [36], Schultz and Alderfer [38], Wood et al [19]
	Information seeking and patients’ actions motivated by viewing results via a portal	Baun et al [50], Giardina et al [28], Henshaw et al [29], Hiremath et al [30], Hulter et al [44], Mangano et al [33], Norris et al [51], Pillemer et al [36], Robinson et al [37], Schultz and Alderfer [38], Wald et al [39], Zhang et al [49]
	Contemplating change in behavior and managing own health	Giardina et al [28], Hulter et al [44], Robinson et al [37], Talboom-Kamp et al [46], Tossaint-Schoenmakers et al [47], Zhang et al [49]
	Benefits of accessing test results via a portal	Baun et al [50], Giardina et al [28], Hulter et al [44], Mák et al [32], Norris et al [51], Pillemer et al [36], Robinson et al [37], Schultz and Alderfer [38], Talboom-Kamp et al [46], Wakefield et al [48]
	Limitations of accessing test results via a portal	Giardina et al [28], Mák et al [32], Robinson et al [37], Schultz and Alderfer [38]
	Suggestions for portal improvement	Baun et al [50], Giardina et al [28], Hulter et al [44], Mák et al [32], Pillemer et al [36], Robinson et al [37], Wald et al [39], Zhang et al [49]
**HCP^a^perspectives (n=7)**		
	Providers’ view of benefits of patient access to results via portal	Henshaw et al [29], Pillemer et al [36], Rodriguez et al [41], Wald et al [39], Winget et al [43]
	Effects on HCP workload	Henshaw et al [29], Pillemer et al [36], Rodriguez et al [41], Wald et al [39], Winget et al [43]
	Concerns about patient anxiety	Henshaw et al [29], Pillemer et al [36], Wald et al [39], Winget et al [43]
	Timing of result release into the patient portal	Henshaw et al [29], Rodriguez et al [41], Winget et al [43]
	The method of result release into the patient portal: manual vs automatic release	Krasowski et al [31], Okawa et al [35], Pillemer et al [36]
	The effects of hospital HIT^b^ on patient quality outcomes	Williams et al [42]

^a^HCP: health care provider.

^b^HIT: health information technology.

The results consist of 2 parts. First, we report patients’ perspectives on accessing their test results via the patient portal, which includes 9 themes. Articles in this group [19,26-40,44-51] analyzed data from patients, family caregivers, and organizational electronic systems. Second, we report HCP perspectives on patient access to test results, which consists of 6 themes. Articles in this group [29,35,36,39,41-43] analyzed data from HCPs and organizational electronic systems.

### Patient Perspectives on Accessing Their Test Results via Portal

#### Theme 1: Patterns of Portal Use and Patient Characteristics

A total of 15 studies [19,26,27,29-31,33,34,36,37,39,​40,45,50,51] included data on varied patterns of portal use. The result release portal feature was popular among patients, especially among outpatient portal users than among patients in the ED. Approximately 70% of 128 surveyed portal users in primary care [39] and 30% [31] to 80% [36] of large samples of portal users in outpatient departments (as seen in the system’s data) viewed test results. In another study, 508 patients viewed 75% of all radiology reports released, with nearly 90% of reports viewed within 1 week [29]. The analysis of outpatient portal logs over a 1-year period showed an average of 13 logins per patient, with nearly half involving a review of test results [36]. Interestingly, another system log analysis showed that approximately 20% of outpatient laboratory results were viewed within 8 hours of release to the patient portal and nearly 10% within 2 hours of release [31]. The researchers concluded that this presents challenges for providers in that some patients may view the results before the provider has had a chance to review the test results in detail or, alternatively, before other results are available [31]. The patients viewed all types of tests available on the portal [37].

In contrast, examination of an electronic data set in the ED at an academic medical center showed that less than 10% of all test results ordered in the ED and released into a patient portal were viewed by patients [27,31], with approximately half accessed within 72 hours [27]. However, Foster and Krasowski [27] noted that this should be considered in conjunction with the portal adoption rate; approximately 37% of all patients seen in the ED during a 1-year study period had an active portal account.

At this academic medical center, in outpatient departments and the ED, patient access rates to their test results were highest among female individuals [27,31], those aged 0 to 11 years (parent or guardian viewing by proxy) and 18 to 60 years, and people who identified as Caucasian or Asian [27]. In contrast, the lowest rates were among teenagers (of note, the organization policy did not allow proxy access for this group) [27,31], those older than 81 years, and people identified as African American, Black, Hispanic, and Latino (these ethnic groups had overall lower rates of portal adoption) [27].

A more recent study [19] conducted at the same medical center but following the implementation of the immediate release of nearly all tests (as opposed to the earlier practice of delayed release for selected tests) found that the viewing results increased significantly for both pediatric and adult patients with existing portal accounts, especially among outpatients. In contrast, there were no significant changes in patients without portal accounts [19]. Researchers have suggested that this indicates increased engagement for existing portal users rather than a widespread increase in portal use [19]. Moreover, the demographic characteristics of portal users in the study by Wood et al [19] were similar to those reported in earlier studies conducted at this medical center [27,31].

A few studies have focused specifically on radiology results. Overall, the vast majority (>88%) of patients felt that releasing radiology reports via the portal was important and wanted to have their own medical images available to them [29,30,51]. Patients who had used a portal before were less willing to wait for results—and more willing to view imaging results online—compared with those that had never used a portal before [40]. In another study, slightly more than half of the 61,131 patients with access to at least one radiology report viewed them, and this positively correlated with viewing laboratory results and clinical notes [34]. Patients express a desire for more comprehensive information, such as inclusion of images to accompany reports [29,33,50], especially for abnormal results [33]. Patients anticipated that the release of imaging results into the portal would eliminate duplicate diagnostic tests [30]. Two studies [29,30] mentioned the participants’ comfort and ease of viewing imaging reports via the portal. Similar to the sociodemographic characteristics of portal users accessing their laboratory results, females viewed radiology reports more often than males [26,30,34]. However, patients who are unfamiliar with portals [40], older [26,34,40], have abnormal results [33], or serious conditions (cancer, cardiovascular disease, and depression) [40,50] use portals less and prefer direct communication with a physician [50].

In one study, patients interacted with radiology reports less frequently than with laboratory reports in the same portal (27% vs 47%). The researchers explained this by easier access to laboratory results that had more patient-friendly features (color coding, labeling, and hyperlinks to explanations) compared with radiology reports [45]. However, there might be another explanation: during the study period, 40% of the patients underwent radiology tests, whereas 61% underwent laboratory tests.

#### Theme 2: Emotional Response When Viewing the Results

A total of 9 studies [28,31,32,36-38,49-51] discussed this aspect of portal use. Viewing laboratory results via a portal may have different emotional effects on patients [28,32,37,38]. However, an overall pattern is that patients feel negative emotions when receiving abnormal results as well as when they are uncertain about how to interpret the results, either because of the results’ implications for their overall health or because the language is unfamiliar. For example, in a sample of 200 surveyed patients, 84% felt positive viewing normal results, whereas only 45% maintained a positive outlook in the presence of abnormal results [49].

In a quantitative analysis, Mák et al [32] compared survey data of portal users (2047) and nonusers and found no significant difference between groups in levels of reported anxiety after receiving test results. Most patients reported little anxiety after receiving laboratory results [32]. This was an ambulatory, view-only web portal serving laboratory services in a Canadian province, and 77% of the participants reported overall excellent or good health despite 60% having chronic conditions and at least 3 laboratory tests in the last 12 months. The researchers indicated that the study design could not differentiate between tests conducted for diagnostic purposes and for monitoring existing and known health condition [32].

Mák et al [32] also examined the relationship between participants’ level of anxiety after accessing results via a portal and the perceived necessity to follow-up with their HCP. Portal users who first learned the results of their most recent laboratory test via a portal and who indicated it was “clear they needed to follow-up were less likely to report no anxiety (38.30%, 95% CI 35.44-41.16) as those who reported not being clear about the need for follow-up (29.84%, 95% CI 24.69-34.98)” [32].

The findings of the interview studies might explain some of the above statistics. In a sample of 95 adults with or without chronic conditions in primary care and a Veterans Affairs facility, 50% felt emotionally neutral when receiving normal test results via a portal [28]. This indifference was attributed to 3 factors: patients had personal medical knowledge about a test, a physician or nurse had called before the patient viewed the results, or a normal result was not of concern [28]. However, viewing abnormal results, more than half of patients felt emotions, such as “confusion, concern, anxiety, fear and frustration” [28]. For patients with cancer, this created a dilemma in whether to review scan results before their scheduled appointments, as the waiting time following “bad result” felt longer and worse [50]. Some patients felt psychological harm from inadvertent manual release of sensitive results that occurred before a provider could discuss the results with them [31]. In another study, half of the caregivers of their children treated for cancer preferred to receive “bad news” directly from the physician or via some type of verbal communication rather than through a portal [38].

Paradoxically, patients can view normal results, but experience negative emotions. Specifically, medical jargon and professional acronyms contribute to confusion [49] and uncertainty in the interpretation of results:

I think some of them [tests] said negative and positive. But then I think for some of the tests that you’re supposed to be positive for an antibody. So, if it says positive on it, you think positive means bad, right?...It said positive and I freaked out, and then I went to talk to my doctor about it.

28

Even when patients understood the wording of the test, negative emotions like anxiety and concern remained when they were unsure about the implications for their overall health, for example, being “afraid the doctor’s going to put me [the patient] on some medications” [28]. In another study, outpatient portal users reported instances when test results caused unnecessary anxiety: an abnormal Pap test caused an outpatient to believe she had cancer, or an elevated blood sugar level caused a patient to be concerned about prediabetes [36]. Notably, the test reports that instigated these concerns were not accompanied by an interpretation [36].

#### Theme 3: Understanding Test Results

Understanding results is an ambiguous phrase with meanings ranging from simple awareness of what the test is evaluating to appreciating behavioral or medication changes that may be required based on the results. In each of the 8 studies comprising this theme [28,32,37,38,44,49-51], patients reported at least some difficulty understanding their test results. Initially learning test results via a portal (as compared with learning from an HCP) was a significant *negative* predictor of comprehension, as were younger age and lower level of education [32]. Test type affects patient understanding: blood tests are easier to understand than radiology reports [38]. One study found a much higher degree of patient understanding, likely due to the familiarity with the tests (most participants had had the test before) [28].

In a sample of 203 participants in the study by Zhang et al [49], more than one-third were unsure whether they understood their laboratory results, could discern normal from abnormal results, or could realize the consequences on their health. Many patients felt confused by “incomprehensible” medical terminology [44,49,50]. In contrast, almost all patients in the study by Giardina et al [28] study knew why a specific test was ordered, more than 80% had this test done before, and more than three-quarters said they understood the test results. Similarly, in another study [51], nearly 80% of participants did not feel confused when viewing their radiology images, and one-third discussed how their understanding increased after viewing the results.

On the portal, patients’ understanding was aided by reference ranges, physicians’ comments [37], and visual cues, such as bolded or flagged values [28]. Every fourth patient asked for a physician’s explanation, some had personal medical knowledge [28], and nearly every second patient [28] searched internet [44]. Caregivers of children diagnosed with cancer learned to understand blood tests within 1-2 months after diagnosis, but radiology results were difficult to understand and required an HCP’s input, especially for abnormal results and in the beginning of a patient’s care trajectory [38].

#### Theme 4: Preferences for Mode and Timing of Result Release

This was one of the least discussed areas in the reviewed studies, with only 2 studies focusing on this subject [38,44] and 4 studies mentioning it briefly [19,28,36,50].

Most patients prefer quick access to their results [38,44]. When in the portal, patients were able to choose between 6 options for when they wanted to receive their results (from 1-day delay to never). More than three-quarters chose a 1-day delay for laboratory results and nearly everyone chose the shortest-available 7-day delay for radiology and pathology results [44]. Similarly, among 19 family caregivers of children with cancer, more than 70% preferred to learn the results as fast as possible (prioritizing speed), whereas others preferred to learn from an HCP versus from the portal (prioritizing mode). Overall, the researchers concluded that “type of testing (radiology/laboratory), the expected result (normal/abnormal) and the time course within their child’s care (closer/further form diagnosis) influenced the preferred mode of delivery” [38]. This study provides a noteworthy example of how clinical diagnosis, participants’ emotions, and their ability to comprehend test results are consequential for their preferences of speed versus mode of result delivery. One US study suggested that national standards for test result release, particularly for sensitive results, are needed [28].

Autoreleasing test results on Friday evenings was problematic for some patients as they had to wait until Monday to call their HCP to clarify the results that worried them [36,50]. However, in a large medical center that recently switched to the immediate release of nearly all laboratory and imaging results, a notable increase in the results released on weekends was observed [19]. This did not change the patients’ viewing patterns, and the researchers concluded that patients view results mostly as convenient to their schedule, regardless of the timing of result release [19].

#### Theme 5: Information Seeking and Patients’ Actions Motivated by Viewing Results via a Portal

As discussed above, flagged test results and physicians’ comments in the portal help patients understand their health information but are not always available or helpful. According to 12 studies [28-30,33,36-39,44,49-51], patients were left needing more information after viewing their results and they searched for this information. Common sources of information include internet free hand search, web-based health forums, family members, or follow-up with their HCPs with a phone call, visiting the clinic, or sending a secure message via the portal [28,29,37-39,44,49-51]. Nearly 50% [28] to 75% of participants [39] viewed reference information hyperlinked on the portal page or conducted internet searches on test results. However, using internet to understand test results was not always helpful, as some participants found the information misleading or disturbing [44,49]. Not knowing whom to ask or feeling embarrassed to ask questions about their images prevented some patients from finding answers to their questions [51].

Even in the presence of a physician’s note explaining results [28,37], participants still searched internet for additional information to look up unfamiliar terminology [37], potential diagnoses, condition-specific information [28], and explanations of trends in laboratory values over time [38]. This could help prepare questions for doctors before their next appointment [36,37,49,50]. More than half of all participants in Giardina et al [28], one-third of participants in Hiremath et al [30], and some in Robinson et al [37] and Norris et al [51] shared or discussed the test results with family or friends.

From 17% [39] to 25% [29] to more than half [51] of patients had questions for, or contacted, their referring HCP for more information after viewing their results, whereas some participants used their results to obtain a second medical opinion [51]. Typically, participants with abnormal results [28] or those viewing radiology reports and images [30] anticipated contacting their physicians. Secure messaging via the portal is a newer way to communicate, which was used by 25% of the patients in Giardina et al [28]. Interestingly, patients who believed their results had not yet been viewed by a physician were more likely to place an in-person or telephone visit than those who believed their results had been reviewed [36]. Some patients hesitate to ask their physicians questions, assuming they are too busy [28,51], or avoid making appointments for what they perceive as minor issues that can be resolved by messaging the physician [37].

#### Theme 6: Contemplating Change in Behavior and Managing Own Health

This theme, supported—to various degrees—by 6 studies [28,37,44,46,47,49], is concerned with how patients consider behavioral and lifestyle changes in relation to their laboratory indicators. For example, patients understand worsening laboratory results as prompts to seek information about lifestyle changes [49] or start medication and change diet [28]. In another study, participants accessing and monitoring their laboratory results scored high on “being encouraged to take health-beneficial actions” [46].

According to the interview data, patients are better able to understand and accept the necessity of lifestyle changes based on laboratory results when they directly observe changes in results. Patient monitoring of their test results can increase awareness and motivation to adopt new practices to improve their health [37,44]. Increased awareness and ownership may be the first step toward positive changes with the intent of maintaining test results within normal reference ranges. “I see the results, I can participate in the results...it makes me feel as if I’m participating more in the overall care of my health” [37].

However, statistical analyses of survey data [46,47] may complicate this picture. In the Netherlands, Talboom-Kamp et al [46] used the eHealth Impact Questionnaire to survey 354 patients who viewed their blood test results via a portal. A significant positive correlation was found between the subscales of Information and Presentation (measuring portal usability) and Motivation and Confidence to Act (*r*_345_=0.77; *P*<.001), indicating greater self-efficacy in patients who were able to navigate the portal and understand their results. Despite rating the usability and presentation of information positively, patients’ self-reported motivation to act on the information was below the set cutoff to indicate a positive result. The authors reason that the low score regarding motivation to act might be a function of the exploratory nature of the study, which was the first to use the eHealth Impact Questionnaire to study the relationship between usability and self-efficacy; thus, they do not have a comparator for what might constitute a positive, negative, or average score on the Motivation and Confidence to Act subscale [46]. In a follow-up study [47] involving 748 patients, self-efficacy tended to be lower among participants with higher education. The authors state that this finding contradicts previous research and note that a low response rate, small sample size, portal design, and extraneous variables may explain this unexpected finding [47].

#### Theme 7: Benefits of Accessing Test Results via a Portal

##### Overview

Ten studies included findings supporting this theme [28,32,36-38,44,46,48,50,51]. Patients reported various degrees of benefits to their health and care, from no change to an increased level of comfort [37], better understanding of personal health, and enhanced confidence to take action [46]. Access to laboratory and imaging results via a portal was seen as progressive and convenient, reducing wait times for results, leading to improved relationships and communication with HCPs, facilitating understanding of health information, and improving engagement in care [28,32,36-38].

##### Convenience and Health Information Archive

The fast availability of results on the portal reduces the wait times to learn one’s results [32,37,38,50] and decreases the need to see the physician in person [37]. Mák et al [32] reported high levels of satisfaction with web-based services, and Schultz and Alderfer [38] found that 58% of caregivers of sick children used the portal either often or sometimes. Participants liked being able to go on the internet and view results because it was easy; they could do it on their own time, especially if they wanted to process “bad” results at home; and come back to it as often as they wanted [37,44]. Moreover, participants liked being able to review their health information and monitor results over time [36-38], and caregivers saw value in being able to keep a record of their child’s history [38]. Participants saved a copy of the results [51] and saw value in future access to and use of the health information archive [38].

##### Relationship and Communication With Physician

Although patients’ beliefs varied, some felt that the portal improved communication [37] and accessibility to their physician [36]. With the ability to view results via a portal and seek additional information, patients were able to prepare [44] and ask further questions at appointments, leaving appointments more satisfied with productive communication [37].

##### Staying Informed and Making Care Decisions

For some patients, the benefits of being informed outweighed their concerns about reading abnormal tests. “Even if the results weren’t good, I’d much rather know. I mean, you cannot have your head in the sand. And I think with as much information as you can, you make better lifestyle decisions” [37]. Participants stated a desire to be stewards of their own health [37] and reported feeling empowered and involved in their treatment and care [50,51]

##### Managing Anxiety Through Fast Access to Information

In the study by Schultz and Alderfer [38], 8 out of 19 caregivers of children diagnosed with cancer indicated that the timely retrieval of results reduced anxiety (it should be noted, however, that any other timely mode of delivery could reduce anxiety). Furthermore, the visualization of trends in laboratory values was important for these parents. For example, seeing that one particular score significantly decreased led to feelings of hope about a child’s prognosis [38]. In other studies, participants experienced reduced anxiety related to not needing to wait for the office to call about results [37,50] and being able to verify that tests were not missed [37].

##### Valuing Independence From Physicians

Despite the observation that patients often want results to be delivered or filtered by a physician [38], they also value independence from physicians’ routines. Rather than being worried about their physician forgetting to notify them, participants experienced peace of mind knowing that they would receive results in a timely manner via a portal and, if necessary, phone to follow-up on their own [37]. This study also found that participants valued the opportunity to view all ordered laboratory results instead of being informed only about clinically significant results [37].

##### Benefits to Health Systems: Linking Fragmented Services

Patients who access their health information via a portal and share it with providers lacking access to those EHRs serve as a link between providers [37,44], a practice rarely acknowledged in the literature, but familiar to patients and clinicians. For example, a participant in the study of Robinson et al [37] printed off magnetic resonance imaging results and shared them with a chiropractor for further explanation of a concern with the spine. Wakefield et al [48] established that among patients with diabetes who accessed 2 portals in the 2 health systems where they were clients, portal use was associated with decreased duplicate glycosylated hemoglobin testing. The researchers suggested that the availability of test results in the portal can facilitate patient sharing of their health information with HCPs, which helps reduce duplication of tests [48].

#### Theme 8: Limitations of Accessing Test Results via a Portal

The reviewed studies described various challenges patients experienced when interacting with the portal, including password issues, displays that were not user-friendly, usability issues [28,39], the necessity to learn new technology [37], and difficulties loading images [51]. These challenges were not universal, as 60% of the participants in Giardina et al [28] did not have any difficulties accessing the results on the portal. Sharing portal log-in information with others (known as unauthorized proxy access) is quite common among patients yet is often described by researchers as problematic. For example, 25% of patients reported that they would share log-in information with their family members [30].

For this review, however, we were interested in challenges or limitations specific to accessing test results via a portal rather than in issues that patients might have with portals overall. Four studies support this theme [28,32,37,38]. The most frequently cited limitation was learning important or “alarming” results before disclosure by the HCP or without appropriate context, which increased the possibility of results being misunderstood and created communications lacking a “personal touch” [37,38]. In fact, initially learning test results via a portal was a significant negative predictor of comprehension [32]. Another issue with learning the results “prematurely” is anxiety and other negative emotions, as discussed in theme 2. Patients also commented on the lack of test result explanations and education (eg, tutorials) in the portals [28,37]. Interestingly, however, even when patients were aware of a reference library in the portal, some did not access it [37].

#### Theme 9: Suggestions for Portal Improvement

In the reviewed articles, participants offered suggestions for improving portals, including increasing the number of health care facilities using the portal so that information is more comprehensive and seamlessly shared [37,44], simplifying navigation in the portal by adding a search function [28], improving usability [39,49], and providing timely test result explanations and follow-up instructions [49]. In addition, some remarks were made regarding meeting the needs of marginalized user groups, such as those with decreased literacy, visually impaired people, and older adults [49].

A total of 8 studies [28,32,36,37,39,44,49,50] included suggestions specific to accessing the test results, the focus of our review. First, participants wanted a notification (eg, an email autosent from the portal to a person’s regular email account) when the results were released to the portal [28]. This was not a universal issue as notifications were a regular feature of patient portals in many health organizations. Second, participants requested that additional results be added to the portal, including radiology reports (x-ray, computed tomography, magnetic resonance imaging, and other imaging), images, and specialty reports to develop a better understanding of their health [28,29,33,37,39,44,49,50]. Next, participants identified the need for portal tools that could help them interpret health information [32,50], such as graphs [36], a graphing feature to track test results over time, or a health encyclopedia functionality [49]. Finally, patients desired physician input on certain forms of written explanations accompanying test results (eg, follow-up instructions and interpretation of the meaning of tests) [36,37], secure messaging with the physician, or the potential for the integration of artificial intelligence technology for the generation of more personalized medical information and health instructions [49].

### Provider Perspectives on Patient Access to Their Laboratory and Imaging Results

#### Theme 1: Providers’ View of Benefits of Patient Access to Results via Portal

Five articles [29,36,39,41,43] addressed HCP perspectives on whether patient access to results via the portal was beneficial, and the findings varied. Some physicians perceived benefits, whereas others, particularly oncologists [41,43], expressed doubts about the benefits, particularly when patient test results were abnormal. Although some physicians appreciated patient access to their test results as a safety measure, for example, when a patient caught an occasional missed result when using a portal [36] or when errors in records were detected by patients and then corrected [39], other physicians were unsure if patient access to test results improved patient safety [36].

Two studies [29,39] showed that physicians perceived clear benefits of patient access to their results via the portal. In a sample of 48 primary and specialty care physicians, nearly 90% agreed that releasing radiology reports to patients was useful [29]. In another small sample of primary care physicians, most suggested that patient access to their results encourages patient engagement in their care [39]. However, these physicians acknowledged the need to improve their current processes for test-result management, such as delays and result-tracking failures. These participants anticipated that patient access and automated systems, such as portal technology, could facilitate test result management [39].

In contrast, oncologists were more skeptical. In a sample of 82 oncologists in an outpatient department at a cancer center, an overwhelming majority felt that web-based patient access to abnormal results had negative consequences, but opinions were mixed for normal results [43]. Approximately half believed that seeing normal results before in-person consultation could be beneficial for patients. In contrast, approximately every third oncologist responded that patients should not see radiology and pathology reports before consultations with their physicians and that result release should only take place if the result delay or embargo period is prolonged [43]. A majority (87%) believed that receiving abnormal or confusing reports before consultation would be harmful for patients, and half of the study participants reported that sharing results via a portal worsened their communication with patients [43]. Several oncologists have emphasized the importance of face-to-face communication to relay sensitive information or bad news because of the need for in-person counseling [43].

In another outpatient cancer center that implemented immediate release of laboratory results into a patient portal, the proportion of physicians and nurses who believed that patients should have access to their laboratory results slightly increased 6-month postimplementation as compared with preimplementation, but this was still about half of the 276 respondents [41].

#### Theme 2: Effects on HCP Workload

Five articles [29,36,39,41,43] addressed the effect of the result release via the patient portal on the HCP workload. Findings were inconsistent both within and across studies, with Rodriguez et al [41] reporting an overall decrease in workload, Winget et al [43] and Pillemer et al [36] reporting an increase in workload, and Henshaw et al [29] and Wald et al [39] reporting mostly no change. Only 2 studies compared the actual numbers of office visits [36] and phone encounters [36,41] pre- and postdirect release of test results, whereas other studies based their analyses on HCP self-reports.

Three articles [29,39,41] reported that the workload remained the same or decreased. More than 70% of 287 nurses and physicians in the outpatient cancer center reported that their workload remained the same or decreased 6 months after implementation of immediate release of laboratory results into the patient portal [41]. The average number of nurses’ phone calls per day during the 3 months after implementation did not change [41]. In another study, among 48 primary care physicians who manually released radiology reports via the portal (timing varied from the same day to >2 wk), 73% reported that their follow-up work (eg, emails, calls, and visits) was unchanged, whereas 13% noted that this work decreased [29]. Further, in a pilot in primary care practices that offered patients access to their laboratory results, feedback from 10 physicians after the first 2 months was mostly positive, with physicians reporting no increase in messages from patients about inconsequential results and no extra time expenditures [39].

In contrast, 2 studies [36,43] reported an increase in the provider workload. In a sample of 82 oncologists in a cancer center, some reported an increased workload [43]: patients’ access to results led to increased communication with patients to provide context, answer questions, and address patient anxiety. This issue was exacerbated when the extra work was not billable [43]. In another study, Pillemer et al [36] evaluated the effects of the release of test results in outpatient facilities of a large health care system over a 1-year period. As per the policy, after the ordering physician views the results, they have the option to manually release the results to the portal. Otherwise, the test results were automatically released within 48 hours. In the interviews, physicians noted differences in the workload between manual versus autoreleased test results, as well as results that require adding physicians’ interpretations versus those that do not [36]. The analysis of portal use by viewers and nonviewers of test results both before and after the automated release showed that viewing test results (by patients) was associated with a small, statistically significant increase in office and telephone visits (3.7% and 4.6%, respectively). The results were similar when analyses were limited to patients who had only normal test findings (3.0% and 4.0%, respectively) and to patients with normal test findings autoreleased (3.9% and 4.9%, respectively; *P*<.001) [36]. However, if physicians manually released test results within the 48-hour embargo period, the increases in office and telephone contact were smaller and not statistically different (1.7% and 2.3% for office visits and phone calls, respectively) [36].

Physicians and oncologists described strategies they used to prevent an increase in follow-up emails and calls. Specifically, they identified the need for appropriate staff support to enable timely response to patients by phone or face-to-face visits [43] and identified the importance of interpretation (eg, by creating ways clinicians can attach a message), ideally added to the portal within the embargo period [36]. Although some physicians suggested writing reports in lay terms so that patients could understand them, most respondents indicated a preference for standardized medical language as best serving system’s needs [29].

#### Theme 3: Concerns About Patient Anxiety

In 4 studies, HCPs were asked about their concerns in relation to patient access to results via the portal, and across these studies, physicians and nurses expressed concerns about the risk of psychological harm, such as patient confusion and anxiety from the direct release of results, especially abnormal results [29,36,39,43]. Occasionally, physicians stop releasing radiology reports because of such concerns [29]. Portal use in these studies varied from 8 weeks [39] to a few years [36]. In all studies, HCPs did not specify whether patients discussed their discomfort and anxiety with them or whether HCPs’ concerns stemmed from their assumptions about patient experiences.

Physicians and nurses linked patients’ increased anxiety to lay people’s inability to understand terminology and interpret test results [43]. HCPs indicated that timing, method (manual vs autorelease), and presence or absence of physician’s interpretation accompanying the tests were related to patient anxiety [29,36,39,43].

Physicians were clear of the necessity to quickly aid patients in interpreting test results to prevent or reduce anxiety [36,39,43]. Secure messaging and result letters have been mentioned as feasible mechanisms to enhance patient interpretation of results [39]. In an organization with a short embargo period (48 h), some physicians noted that manual release of the results within the embargo period helped eliminate patient anxiety [36]. Only one study [31] provided specific details about EHR adjustments undertaken to reduce the chance of HCPs inadvertently releasing abnormal results before in-person discussions with a patient, such as a more prominent display of the timing of release or adding a button to mark results as reviewed in the EHR without manually releasing them to the portal.

#### Theme 4: Timing of Result Release Into the Patient Portal

Three articles [29,41,43] addressed HCP perspectives on the timing of result release into the patient portal. Overall, the HCPs in these studies were in consensus about their dislike of *immediate* autorelease of test results, especially “sensitive results” [29,41,43].

In primary and specialty care clinics where referring HCPs (mostly physicians) had the option of manually releasing reports, they released 53% of reports to patients on the same day reports became available, 36% within a week, and 11% 2 or more weeks after reports became available [29]. However, when offered the *autorelease* of reports following *a 7-day delay period*, as many as 42% of physicians still disagree with this option [29]. Autorelease of x-ray reports, with a 1-week delay, was preferred by 58%, but they were more reluctant to autorelease computed tomography and magnetic resonance imaging reports [29].

In an outpatient oncology center, the portal policy stated that patients would receive an email to let them know that their results were available in the portal upon physician review and approval of results or 7 days after the report was finalized, whichever came first [43]. This embargo period was designed to allow clinicians time to review and communicate the results with patients. As many as one-third of oncologists believe that patients should never see results before consultations with primary physicians, stressing the importance of meeting patients face-to-face to first relay bad news or sensitive information [43]. Nearly 90% of respondents said that the expected effects on patients who received abnormal results before physician consultation would be harmful [43]. Oncologists generally supported a minimum 7-day embargo period [43].

The above findings were amplified by those from another oncology center that implemented the immediate release of laboratory results [41]. Although 6 months after implementation more nurses and physicians became comfortable with patients’ immediate access (as compared with preimplementation), as much as 40% to 48% of 281 respondents still felt uncomfortable about immediate release [41]. Most respondents felt that the patients could not interpret their results [41].

#### Theme 5: The Method of Result Release into the Patient Portal (Manual vs Automatic Release)

Varying levels of detail concerning the method of result release were provided in the reviewed articles, from a brief mention of the method to a detailed information on institutional policies (or result release framework) guiding whether and what results were autoreleased, either immediately or after a delay, versus released manually [31,35,36]. Moreover, none of the reviewed articles indicated whether HCPs were consulted during the process of policy development or how much control physicians had over the method and timing of result release in their individual practice.

The associations between the method of release, patient anxiety, and HCP workload are addressed in the corresponding sections. The autorelease of test results into patient portals leads to a much higher volume of reports accessible to patients. In contrast, with the manual release policy, physicians release results only selectively.

When Kaiser Permanente Hawaii transitioned from the manual release of imaging reports to an autorelease on day 3, reports available to patients within a week increased from 82% in 2013 with manual release to almost 99% in 2015 with autorelease [35]. The total number of reports available in the patient portal increased 6-fold. In 2015, reports were released for 52,293 patients, 56% of whom were active on the patient portal. Regardless of the enhanced availability, patients tended to access the same proportion of the results. One explanation offered by the researchers is that more than 70% of the results that were not accessed by patients were released to patients inactive on the portal [35].

The study by Krasowski et al [31] reported approximately equal ratios of autoreleased and manually released reports of chemistry and hematology tests (most of which had a 1-day embargo) in outpatient settings (where manual release was technically easy). For most other test categories, such as microbiology, anatomical pathology, x-ray, and magnetic resonance imaging (most of which had a 4-day embargo), manual release was more common [31]. Across all test categories, 30% to 55% of the results were released to the portal within 12 h of first appearing in the EHR [31].

Different patterns were observed in the ED, where most results were autoreleased (except anatomical pathology and microbiology, which had the highest frequency of manual release within 2 h of being available) [31]. One reason the researchers offered is that manual release was less streamlined in the ED and required more clicks on the computer screen [31]. These results indicate a relationship between the method of result release, the complexity of tests, and the clinical context that health organizations consider when creating portal policies.

The researchers observed that the variability in result release (manual vs autorelease, next day vs 4 days) can be confusing for both patients and HCPs [31]. To address these concerns, HCP education was developed, and the column was added to the EHR to notify the planned release date, but this remains challenging with regular HCP turnover [31].

#### Theme 6: The Effects of the Hospital Health Information Technology System on Patient Quality Outcomes

One study contributed data on the correlation between health information technology (HIT) systems and patient quality outcomes. Williams et al [42] examined 1039 medical and surgical hospitals in the United States and their HIT systems to analyze the correlation between the level of technology (among other variables) and patient outcomes. The quality predictor variables were staffing levels, hospital size, and HIT, such as patient health records, computerized provider order entry, and HCP electronic access to diagnostic results. Patient outcome variables included hospital readmission and mortality rates [42].

Researchers [42] found that HIT providing HCPs with electronic access to diagnostic results was the most influential technological characteristic affecting patient quality outcomes. For analysis, a quality score of 1 was set as the target variable. The number of full-time equivalents, staffed beds, and type of HIT were set as predictor variables. The results suggest that the number of staffed beds is the most influential variable in the model overall (100% correlation). HCP electronic access to diagnostic results (18.427% correlation) was the most influential technological characteristic of hospitals that had a quality score of 1, although the association was weak. In other words, HCP electronic access to diagnostic results, although a weak predictor of patient outcomes in the model as a whole, was the most important variable among HIT applications. The study also found that 70% of hospitals with quality scores closest to 1 used only 1 HIT application, whereas 45% of hospitals with quality scores less than 1 used 2 or more HIT applications [42].

The implication is that more technology does not necessarily improve the quality of patient care; hospital size and staffing levels are more indicative of hospital quality [42]. The authors concluded that as technology is an adjunct to the process of providing care and not the sole solution, decision makers should consider other quality needs before adopting technology [42].

## Discussion

### Summary of Key Findings

In this scoping review, we examined 27 research studies reporting patient and HCP perspectives on patient web access to their laboratory and imaging test results using patient portals. Most of the studies were published after 2016 and originated in the United States. Among the themes comprising patient perspectives, 3 were addressed in 10 or more articles: demographic factors associated with portal use, information seeking to clarify results, and benefits of viewing test results. In contrast, 2 themes—the mode and timing of result release and behavioral changes motivated by viewing one’s test results—were addressed in 6 articles. Only 7 articles included HCP perspectives or analyzed the patterns of result release. Among the themes comprising HCP perspectives, changes in the workload and HCP perception of benefits of patient access to results were addressed the most, whereas the mode and timing of result release and concerns about patient anxiety were addressed the least. Only one article analyzed patient quality outcomes in relation to hospitals’ HIT, including HCPs’ access to test results in the electronic records. The findings of our review are too numerous to be summarized in their entirety. Several findings, namely, sociodemographic factors of patients who view their results or mixed effects on HCP workload, rehearse well-known findings from other studies. Thus, we focus on the selected findings.

An important but underrecognized benefit of patient access to their test results is that in the reality of fragmented health services, portals serve as archives [36-38] and assist patients to act as an information link between service providers who do not have access to the same clinical information systems [37].

Patient anxiety is a shared theme across the patient and HCP perspectives. Our findings show that 2 main factors contribute to patients’ negative emotions and anxiety: viewing abnormal or incomprehensible test results. Each of these factors seems to be mediated (albeit in various ways and to various degrees) by the mode (manual vs autorelease), timing of result release (immediate vs delayed), and whether the latter permits timely direct communication between patients and HCPs (text, phone call, and visit) either before or shortly after the result is released into the portal.

An observation that can be drawn from the reviewed studies is that the timing of the result release is important for all stakeholders. For patients, the timing was consequential for how they felt about and what steps they took in response to these results. Both “too early” and “too late” availability of test results was capable of generating anxiety, producing a flurry of actions to seek additional information, and altering patients’ comprehension of test results. HCPs in several studies have conveyed their dislike for immediate release [29,31,41,43]. The concern was that patients may view the results before the provider has had a chance to review them in detail or, alternatively, before other results are available [31]. Oncologists generally supported no less than a 7-day embargo period [43].

Considering the ubiquity of references to *time* among patients*,* it was surprising that studies reporting patients’ views rarely provided detailed information about the organizational result-release framework. One explanation is that patients themselves were not always sure when the results were released, as can be seen in Robinson et al [37]. Only a limited number of articles included detailed background information on health organizations’ result release frameworks, including timing [27,31,35,36].

To mitigate patient anxiety, aid patients’ comprehension of results, and prevent unwarranted increases in HCP workload, 3 main strategies have been suggested in the reviewed studies. First, ordering HCPs should educate patients about the purpose and potential findings of the tests being ordered when the tests are ordered. Patients did not experience negative emotions when viewing their results because the physician explained why the tests were being performed when they were ordered, and half of the participants were told to check the portal for their results [20]. However, this proactive patient education, although helpful, did not address all the limitations that patients face when viewing test results via portals [28].

The second strategy is to enhance the visual display of test results, which was mentioned as patients’ suggestions for portal improvement. The third strategy is to add written interpretations of the test results released into a portal. Patients liked being able to rely on physician annotations and interpretations; knowing that the physician was looking at the results relieved concern and anxiety. However, despite the HCP’s awareness of the benefits of providing written commentary, this was not commonly done, leaving patients searching for alternative sources of information, including the internet and family members.

Although it is important for health organizations to implement EHRs and support HCPs’ access to test results to support treatment decisions, more ICT does not necessarily improve the quality of patient care, as hospital size and staffing levels can have greater effects on reducing patient readmission rates and perceived quality of care [42]. This point is echoed in the broader portal literature that patients do not consider portals to replace human interaction but rather complement it [54,56].

### Comparison With Other Literature

Our findings support some better-established themes in research on patient portals, while expanding other themes. For example, Antonio et al [2] reported that one of the most widely used and useful portal features is access to the test results. At the same time, patients consistently notice that medical terminology creates challenges in comprehending test reports and that electronic links to credible reference materials might help [2].

In efforts to enhance patients’ ability to interpret test results in a portal, various studies examined whether the format for result presentation influences patients’ understanding, perception of risk, and the follow-up steps [24,25,57]. This literature corroborates our findings related to patients’ difficulties in interpreting the test results. In a controlled study, Fraccaro et al [24] found that 20 kidney transplant patients (mean age 52 y, knowledgeable about their disease, relatively high level of education, regular computer users, and mostly portal users) viewing hypothetical scenarios related to their health condition had difficulty estimating the gravity of laboratory results and whether or not action was required, particularly in response to medium-risk results (compared with high or low risk). As many as 65% of participants underestimated the need for action at least once [24]. Contrary to what might have been expected, the accuracy of the interpretation and estimation of risk did not improve with visual and graphical cues and other variations in the result presentation [24]. Some solutions include patient education before using a portal and integrating the natural tendency to view information (left to right and top to bottom) to display important information and avoid information overload [24]. Other recommendations in the literature include adding a brief written explanation to accompany graphs, tables, and charts; indicating the level of urgency rather than simply a deviation from the norm; and including in the report the type of follow-up required [25]. Furthermore, stating the level of variation that is considered clinically significant may assist patients in delineating which laboratory values are of concern as opposed to fluctuations within an acceptable range [57]. Individualizing the frame of reference to the patient’s age, condition, or other factors and providing thresholds for concern would make the results more meaningful [57]. Ultimately, however, patients’ difficulties in interpreting test results raise patient safety concerns and suggest limits to portal potential [24].

Patient characteristics associated with viewing web-based test results that we found in our review (predominantly female, White, and higher level of education) are also recognized in the literature [2,58]. In addition, research into patient portal use is typically in consensus that health literacy and comfort with navigating health care technology is higher in young adults (aged 25-39 y). However, this should be taken with caution to avoid agist assumptions, as other studies found that the medical necessity to track one’s test results, especially for oncology patients, motivates older people (aged >70 y) to use the portal [54].

Similar to the study by Pillemer et al [36], Alpert et al [59] discussed increased patient anxiety as a significant deterrent of releasing results before a physician has a chance to review them or follow-up with the patient to explain and answer questions; however, many groups of patients prefer quick results release. One example is patients diagnosed with cancer, among whom portal uptake and access to test results are high [60]. Phadke et al [61] found that patients with breast cancer prioritize obtaining results as quickly as possible; however, specific patient communication preferences differ depending on age, cancer stage, and education level. Arenson [62] found that patient preference for the method of receiving results varied, but immediate release into a portal was preferred if it was followed by a prompt call or office visit. Evidently, timely access to test results is particularly important for people with cancer. Patients are interested in viewing clinical information via a portal despite potential occasional “concerns of loss of patient confidentiality, health information inaccuracy, and disruption of the current patient–doctor relationship” [34].

Our discussion of patient information-seeking behavior has tapped into the growing scholarly field of health literacy. Unlike publications in this field [63-65], the studies we reviewed did not focus on patients’ strategies for evaluating the trustworthiness of web-based sources of medical information.

Our findings confirm that providing patients with laboratory results allows them to monitor their results, provides them with additional time to find information specific to certain conditions, and helps them prepare questions for HCPs based on the results [54,66]. In contrast, the complexity of the link between patient access to personal health data, changing health behaviors, and achieving clinical improvements is well recognized in the portal literature [2,67]. This is reflected in our findings, whereby the theme of patients pursuing behavioral changes and self-monitoring their health by observing trends in laboratory values was one of the least saturated in our review.

### Recommendations for Practice and Policy

Efforts to implement patient portals should mitigate the potential of technology to widen health disparities [20] and advocate policy changes to help those with lower socioeconomic status gain access [68]. HCPs can help engage patients in their care and promote patient autonomy and informed decision-making by understanding patient access to test results via a portal and, when necessary, guiding patients on how to sign-up and use portals. However, portal implementers and health organizations should also support physicians and nurses by ensuring that they are well aware of this technology and have the time to teach patients. Encouraging patient portal use has additional benefits, as in some cases (eg, travel or emergencies), patients can provide their medical history via access to their home portal. This provision would also allow HCPs, both internal and external to the patient’s usual health care network, to avoid duplicate testing.

In primary and outpatient health settings, nurses are often assigned the role of triaging patient messages received via portals; however, this contribution often remains invisible in the literature. Nurses can capitalize on their expertise and negotiate a more structured role in explaining results to patients and answering patient concerns and questions about the results. Nurses can triage portal messages and decide whether follow-up appointments are necessary, thus benefiting patients and the health system. These nurses’ roles should be formally recognized rather than remaining invisible ad hoc arrangements. To mitigate the issue of patient anxiety arising from accessing uninterpreted test results in real time, nurses can collaborate with physicians to ensure that patients are aware of the purpose of the tests, the timing of result release, anticipated findings, and further steps. Timely and adequate communication between HCPs and patients is the key to the successful implementation of a direct result release via portals, and nurses should be recognized as important health care team players able to support this communication process and patient outcomes.

As the reviewed studies demonstrate, the result release framework (ie, the policies on the timing and method of result release in a particular organization) is an obscure “background” that influences HCPs’ work and patients but is rarely explicitly acknowledged. It would be beneficial for HCPs to be aware of the timing and method of result release to explain the process of receiving results to patients. Understanding the result release framework in their organizations can also assist HCPs in contributing to policies and procedures for result releases. Clinicians working alongside informaticians ensure that ICTs are configured and implemented in such a way as to support care processes rather than create unintended obstacles. They can also recommend systems that include the interpretation of results that are dictated in lay terminology instead of only medical terminology that patients often do not understand.

It is important to consider the embargo period in relation to the patient population, physician specialty, and type of results being released. Methods of result notification should suit the environment and work practices of HCPs while meeting patient needs. It is also important to involve clinicians (eg, physicians and nurses) when considering options for manual versus autorelease, which can have implications for provider adoption of technology. However, adherence to the Cures Act might mean that health organizations in the United States are switching to immediate release without adequately assessing the consequences for patient safety.

### Recommendations for Research

Most studies included in this review reported the first few months following the implementation of direct result release into the patient portal. Future research can report longitudinal data on how provider attitudes and practices change when supporting patients viewing test results via a portal. New studies can elucidate strategies by which health care settings deal with the consequences of direct result release (eg, what new roles emerge within teams to support patient inquiries). Crucially, future research should provide details about not only the type of portals, but also the result release frameworks and other organizational policies guiding direct result release to the portal. Few studies have analyzed the factors and processes related to organizational decisions in result release frameworks. Furthermore, it is unclear whether and how clinician stakeholders (especially less visible stakeholders such as nurses) are involved in decision-making processes. Another interesting direction arises from the observation that patients are conscious about “not disrupting clinic flow” when weighing the necessity for a follow-up to clarify test results in the portal. Future studies could focus on how portals and patient access to test results affect health-service provision.

### Limitations and Strengths

The data available for this review were restricted to data in the included articles. Although we conducted a systematic search, there is a possibility that not all relevant studies were retrieved. The inclusion of gray sources, articles not written in English, and those addressing low-resource countries might have produced different results. Most of the included studies were performed in the United States. In contrast, the degree of contextual similarities across the included studies (high-resource countries, similar portal technology, and including the EPIC patient portal in several studies) provides stronger support for our findings. Although no formal quality appraisal was completed (in line with the scoping review methodology), we paid attention to methodological rigor and explicitly reported methodological details consequential to the interpretation of the findings. For instance, some authors noted poor response rates despite having adequate numbers of participants or that sample sizes or sociodemographic composition may have affected the results.

The strengths of this review include the separate reporting of patient and HCP perspectives. Although many primary research studies and reviews address patient portals overall, and access to test results is one of the most (or most) used portal features, there were no published reviews *focusing* on patient access to test results via portals. Furthermore, all findings in this review are contextualized by providing details about health settings, clinical specialty, portal type, and the result release framework based on the information available in the primary studies. This information is necessary to develop an understanding of what works for whom, where, and in what circumstances.

### Conclusions

The results of this review illuminate patient and HCP perspectives on patient access to laboratory and imaging test results via a portal. The findings emphasize that all stakeholders have an interest in patient access to timely and comprehensible test results, yet there are challenges. Both patients and providers recognize the many opportunities to improve communication, patient engagement, and relationships with the health care team through the introduction of patient portals. Critical to this desire is support for patients via resources such as provision of normal laboratory ranges, physician comments, and an opportunity to discuss results with a health professional in a timely manner to decrease anxiety or confusion. Health organizations and portal administrators must also consider the sensitive nature of certain results (eg, in oncology), as patients may be especially anxious to receive and interpret the tests and their consequences on health. Decisions regarding the result-release framework require the involvement of clinicians. With the increasing implementation of patient portals internationally, our findings can inform various stakeholders about how to make test releases via portals that support patients and providers.

## Appendix

Multimedia Appendix 1 Search strategy and inclusion criteria.

Multimedia Appendix 2 PRISMA checklist.

## Abbreviations

ED: emergency department
EHR: electronic health record
HCP: health care provider
HIT: health information technology
ICT: information and communication technology
PRISMA: Preferred Reporting Items for Systematic Reviews and Meta-Analyses
PRISMA-ScR: Preferred Reporting Items for Systematic Reviews and Meta-Analyses extension for Scoping Reviews
