# Supplementary material for: Patient and Health Care Provider Perspectives on Patient Access to Test Results via Web Portals: Scoping Review
Source: J Med Internet Res. 2023 Oct 19;25:e43765. doi: 10.2196/43765 (PMC10623227; doi:10.2196/43765)
Supplement: Multimedia Appendix 1 [file jmir_v25i1e43765_app1.docx]

**Multimedia Appendix 1**

**Search Strategies and Inclusion/Exclusion Criteria – May 2020**

| Medline OVID 1946 - May 21, 2020 | | |
| --- | --- | --- |
| Date of search: May 22, 2020 | | |
| Line # | Search Terms | # of Results |
| 1 | patient portals/ | 355 |
| 2 | ((patient* or consumer*) adj2 portal*).ti,ab,kf. | 4,660 |
| 3 | (portal hypertension or portal vein* or portal venous or ((proton or carbon) adj2 portal*)).mp. | 53,686 |
| 4 | 2 not 3 | 1,437 |
| 5 | 1 or 4 | 1,536 |
| 6 | (exp "Health Records, Personal"/ or "Patient Access to Records"/) and (electronic or online or internet or web or portal* or tethered).mp. | 1,579 |
| 7 | ((tethered adj3 record*) or eclinician or mychart).ti,ab,kf. | 108 |
| 8 | 1 or 5 or 6 or 7 | 2,647 |
| 9 | Diagnostic Tests, Routine/ | 12,046 |
| 10 | exp Clinical Laboratory Techniques/ | 2,573,584 |
| 11 | exp Diagnostic Imaging/ | 2,622,897 |
| 12 | ((laboratory or diagnostic) adj (test* or procedure? or technique? or result? or medicine or service?)).ti,ab,kf. | 140,848 |
| 13 | 9 or 10 or 11 or 12 | 4,999,450 |
| 14 | 8 and 13 | 240 |

| EMBASE 1974 to May 21, 2020 | | |
| --- | --- | --- |
| date of search: May 22, 2020 | | |
| Line # | search term | results |
| 1 | electronic health record/ | 16,197 |
| 2 | ((patient* or consumer*) adj2 portal*).ti,ab,kw. | 6,930 |
| 3 | ("portal hypertension" or "portal vein*" or "portal venous" or ((proton or carbon) adj2 portal*)).mp. | 87,676 |
| 4 | 2 not 3 | 1,950 |
| 5 | 1 or 4 | 17,853 |
| 6 | (medical record/ or patient right/) and (electronic or online or internet or web or portal* or tethered).ti,ab,kw. | 13,930 |
| 7 | ((tethered adj3 record*) or eclinician or mychart).ti,ab,kw. | 189 |
| 8 | 5 or 6 or 7 | 30,874 |
| 9 | diagnostic test/ | 77,943 |
| 10 | exp laboratory technique/ | 163,540 |
| 11 | diagnostic imaging/ | 180,958 |
| 12 | ((laboratory or lab or diagnostic) adj (test* or procedure? or technique? or result? or medicine or service?)).ti,ab,kw. | 212,772 |
| 13 | 9 or 10 or 11 or 12 | 562,321 |
| 14 | 8 and 13 | 1,381 |

| CINAHL Plus with full text | | |  |
| --- | --- | --- | --- |
| Date of Search: May 22, 2020 | | |  |
| Line # | Search Term | # results | |
| S1 | (MH “Patient Portals”) | 88 | |
| S2 | TI ( ((patient* or consumer*) N2 portal*) ) OR AB ( ((patient* or consumer*) N2 portal*) ) | 1,455 | |
| S3 | TI ( (portal hypertension or portal vein* or portal venous or ((proton or carbon) N2 portal*)) ) OR AB ( (portal hypertension or portal vein* or portal venous or ((proton or carbon) N2 portal*)) ) | 5,000 | |
| S4 | S2 NOT S3 | 952 | |
| S5 | S1 OR S4 | 983 | |
| S6 | (MH "Medical Records, Personal") OR (MH "Patient Access to Records") OR (MH "Electronic Health Records+") | 27,854 | |
| S7 | TI ( ((tethered N2 record*) or eclinician or mychart) ) OR AB ( ((tethered N2 record*) or eclinician or mychart) ) | 47 | |
| S8 | S1 OR S5 OR S6 OR S7 | 28,439 | |
| S9 | (MH "Diagnostic Tests, Routine") | 4,170 | |
| S10 | (MH "Clinical Laboratories+") | 6,524 | |
| S11 | (MH "Diagnostic Imaging+") | 457,011 | |
| S12 | ((laboratory or diagnostic) N1 (test* or procedure? or technique? or result? or medicine or service?)) | 43,258 | |
| S13 | S9 OR S10 OR S11 OR S12 | 488,498 | |
| S14 | S8 AND S13 | 1140 | |

| Cochrane | | |
| --- | --- | --- |
| Date of search: May 14, 2020 | | |
| Line # | Search Terms | # of Results |
| #1 | [mh ^"patient portals"] | 14 |
| #2 | ((patient* or consumer*) NEAR/2 portal*):ti,ab,kw | 574 |
| #3 | (portal hypertension or portal vein* or portal venous or ((proton or carbon) NEAR/2 portal*)).mp. | 16,136 |
| #4 | #2 NOT #3 | 571 |
| #5 | #1 OR #4 | 571 |
| #6 | [mh "Health records, personal"] OR [mh ^"Patient access to records"] AND (electronic or online or internet or web or portal* or tethered).mp. | 16,206 |
| #7 | ((tethered NEAR/3 record*) or eclinician or mychart):ti,ab,kw | 31 |
| #8 | #1 OR #5 OR #6 OR #7 | 16,775 |
| #9 | [mh ^"diagnostic tests, routine"] | 224 |
| #10 | [mh "clinical laboratory techniques"] | 42,825 |
| #11 | [mh "diagnostic imaging"] | 46,415 |
| #12 | ((laboratory or diagnostic) NEAR/1 (test* or procedure? or technique? or result? or medicine or service?)):ti,ab,kw | 19,768 |
| #13 | #9 OR #10 OR #11 OR #12 | 104,598 |
| #14 | #8 AND #13 | 1,521 |
|  | Trials Not Included | 1314 |
|  | Editorials Not Included | 2 |
|  | Reviews Included | 172 |
|  | Protocols Included | 33 |
|  | Total Imported | 205 |

| Scopus | | |
| --- | --- | --- |
| Date of search: May 22, 2020 | | |
| 1 | ( TITLE-ABS-KEY ( ( patient W/2 portal* ) ) AND TITLE-ABS-KEY ( ( ( patient* OR consumer* ) W/2 portal* ) ) ) | 7,935 |
| 2 | TITLE-ABS-KEY ( ( portal AND hypertension OR portal AND vein* OR portal AND venous OR ( ( proton OR carbon ) W/2 portal* ) ) ) | 19,122 |
| 3 | ( ( TITLE-ABS-KEY ( ( patient W/2 portal* ) ) AND TITLE-ABS-KEY ( ( ( patient* OR consumer* ) W/2 portal* ) ) ) ) AND NOT ( TITLE-ABS-KEY ( ( portal AND hypertension OR portal AND vein* OR portal AND venous OR ( ( proton OR carbon ) W/2 portal* ) ) ) ) | 6,051 |
| 4 | TITLE-ABS-KEY ( "patient portal*" ) | 1,352 |
| 5 | ( ( ( TITLE-ABS-KEY ( ( patient W/2 portal* ) ) AND TITLE-ABS-KEY ( ( ( patient* OR consumer* ) W/2 portal* ) ) ) ) AND NOT ( TITLE-ABS-KEY ( ( portal AND hypertension OR portal AND vein* OR portal AND venous OR ( ( proton OR carbon ) W/2 portal* ) ) ) ) ) OR ( TITLE-ABS-KEY ( "patient portal*" ) ) | 6,121 |
| 6 | ( TITLE-ABS-KEY ( "Health records, personal" OR "patient access to records" AND ( electronic OR online OR internet OR web OR portal* OR tethered ) ) AND TITLE-ABS-KEY ( ( ( tethered W/3 record* ) OR eclinician OR mychart ) ) ) | 17 |
| 7 | ( ( ( TITLE-ABS-KEY ( ( patient W/2 portal* ) ) AND TITLE-ABS-KEY ( ( ( patient* OR consumer* ) W/2 portal* ) ) ) ) AND NOT ( TITLE-ABS-KEY ( ( portal AND hypertension OR portal AND vein* OR portal AND venous OR ( ( proton OR carbon ) W/2 portal* ) ) ) ) ) OR ( TITLE-ABS-KEY ( "patient portal*" ) ) OR ( ( ( ( TITLE-ABS-KEY ( ( patient W/2 portal* ) ) AND TITLE-ABS-KEY ( ( ( patient* OR consumer* ) W/2 portal* ) ) ) ) AND NOT ( TITLE-ABS-KEY ( ( portal AND hypertension OR portal AND vein* OR portal AND venous OR ( ( proton OR carbon ) W/2 portal* ) ) ) ) ) OR ( TITLE-ABS-KEY ( "patient portal*" ) ) ) OR ( ( TITLE-ABS-KEY ( "Health records, personal" OR "patient access to records" AND ( electronic OR online OR internet OR web OR portal* OR tethered ) ) AND TITLE-ABS-KEY ( ( ( tethered W/3 record* ) OR eclinician OR mychart ) ) ) ) | 6,130 |
| 8 | ( TITLE-ABS-KEY ( "Diagnostic rests, routine" ) OR TITLE-ABS-KEY ( "clinical laboratory techniques" ) OR TITLE-ABS-KEY ( "Diagnostic imaging" ) OR TITLE-ABS-KEY ( ( ( laboratory OR diagnostic ) W/1 ( test* OR procedure? OR technique? OR result? OR medicine OR service? ) ) ) ) | 956,487 |
| 9 | ( ( ( ( TITLE-ABS-KEY ( ( patient W/2 portal* ) ) AND TITLE-ABS-KEY ( ( ( patient* OR consumer* ) W/2 portal* ) ) ) ) AND NOT ( TITLE-ABS-KEY ( ( portal AND hypertension OR portal AND vein* OR portal AND venous OR ( ( proton OR carbon ) W/2 portal* ) ) ) ) ) OR ( TITLE-ABS-KEY ( "patient portal*" ) ) OR ( ( ( ( TITLE-ABS-KEY ( ( patient W/2 portal* ) ) AND TITLE-ABS-KEY ( ( ( patient* OR consumer* ) W/2 portal* ) ) ) ) AND NOT ( TITLE-ABS-KEY ( ( portal AND hypertension OR portal AND vein* OR portal AND venous OR ( ( proton OR carbon ) W/2 portal* ) ) ) ) ) OR ( TITLE-ABS-KEY ( "patient portal*" ) ) ) OR ( ( TITLE-ABS-KEY ( "Health records, personal" OR "patient access to records" AND ( electronic OR online OR internet OR web OR portal* OR tethered ) ) AND TITLE-ABS-KEY ( ( ( tethered W/3 record* ) OR eclinician OR mychart ) ) ) ) ) AND ( ( TITLE-ABS-KEY ( "Diagnostic rests, routine" ) OR TITLE-ABS-KEY ( "clinical laboratory techniques" ) OR TITLE-ABS-KEY ( "Diagnostic imaging" ) OR TITLE-ABS-KEY ( ( ( laboratory OR diagnostic ) W/1 ( test* OR procedure? OR technique? OR result? OR medicine OR service? ) ) ) ) ) | 357 |

| Inclusion Criteria | Exclusion Criteria |
| --- | --- |
| 1. Phenomenon of interest: patients accessing their lab/imaging diagnostic test results online (effects on patients or care givers) 2. Population: Patients; family care givers 3. Technology: tethered patient portals, personal health records that allow patient access to lab/imaging results, or online/web portals for patients accessing test results. Actual, existing portals; and usability testing of actual portal prototypes in clinical settings. 4. Primary research of any design 5. High resource countries | 1. Not focused on patient’s perspectives (rather on health care providers’ perspectives and health organizations’ views on patient access to lab results) 2. Not about patients accessing lab/imaging results online 3. Tangentially mentions % of patients or that patients view lab results via portals but main focus is different; or mentions patients hypothetically interested in viewing lab results 4. Genetic portals and inpatient portals for hospitalized patients 5. Broader software development and implementation (not result release) 6. Wrong technology: not portals at all;   portal not tethered but requiring manual entry by third party (researchers)   1. Middle and low resource countries 2. Review papers 3. Preliminary study for which complete study exists, trial registration   10)Grey literature: Book chapters, conference proceedings, op-ed, letters, government or other reports  11)Full text not available |

**Search Strategies and Inclusion/Exclusion Criteria – September 2020**

Two databases: MEDLINE and CINAHL

S1 TI (patient* or consumer*) N2 portal* OR AB (patient* or consumer*) N2 portal*)

S2 TI (“portal hypertension” or “portal vein*” or “portal venous” or ((proton or carbon) N2 portal*)) OR AB (“portal hypertension” or “portal vein*” or “portal venous” or ((proton or carbon) N2 portal*)))

S3 S1 NOT S2

S4 TI (electronic or online or internet or web or portal* or tethered) AND “health record*” OR AB ((electronic or online or internet or web or portal* or tethered) AND “health record*”)

S5 TI ((tethered N3 record*) or eclinician or mychart) OR AB ((tethered N3 record*) or eclinician or mychart))

S6 S1 or S3 or S4 or S5

S7 TI ((laboratory or lab or diagnostic) N1 (test* or result*)) OR AB ((laboratory or lab or diagnostic) N1 (test* or result*)))

S8 S6 AND S7

Search parameters: English language, publication date from 2005-2020 and academic journals.

Search was limited to title and abstract as indicated in search above; title (TI) OR abstract (AB).

The full search was completed in CINAHL database first, which yielded 305 results.

The full search was then completed in MEDLINE database second, which yielded 584 results.

Therefore, the total result of this initial search is 889 results.

| Inclusion Criteria | Exclusion Criteria |
| --- | --- |
| 1). Phenomenon of interest: health care providers’ perspectives and health organizations’ considerations on patient access to lab/ imaging diagnostic test results online  2). Technology: tethered patient portals, personal health records that allow patient access to lab/imaging results, or online/web portals for patients accessing lab results (in primary care and outpatient settings). Actual, existing portals; and usability testing of portal prototypes designed for actual clinical usage.  3). High resource countries (e.g., US, Canada, UK, Netherlands)  4). Primary research of any design | 1).Wrong phenomenon: patients’ and families’ perspectives on accessing their lab/imaging diagnostic test results online  2). Not about patients accessing lab/imaging results online  3). Tangentially mentions % of patients or that patients view lab results via portals but main focus is different; or mentions hypothetical access  4). Broader software development and implementation (not result release)  5). Wrong technology: not portals at all; portal not tethered but requiring manual entry by third party (researchers); inpatient portals; genetic portals  6). Context: low-resource countries  7). Reviews, preliminary study for which complete study exists, trial registration  8). Grey sources: Book chapters, conference proceedings, op-ed, letters  9). Full text not available |

**Search Strategies and Inclusion/Exclusion Criteria – August 2023**


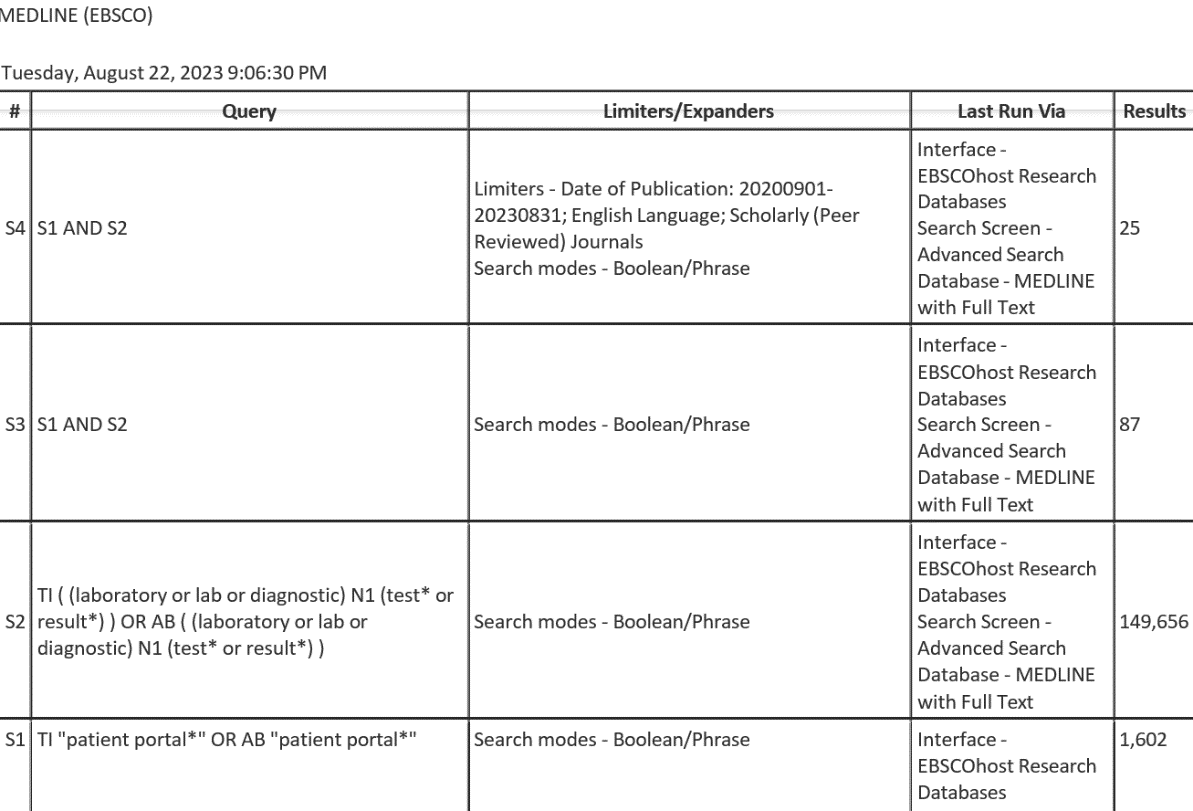

| Inclusion Criteria | Exclusion Criteria |
| --- | --- |
| 1) phenomenon of interest: patients accessing their lab or imaging diagnostic test results online via patient portals;  2) population: patients and/or family caregivers, healthcare providers, health systems;  3) technology: tethered patient portals, personal health records that allow patient access to lab/imaging results, or online/web portals for patients accessing lab/imaging results. We were interested in actual, existing portals; or usability testing of actual portal prototypes designed for clinical settings;  4) the type of publications: primary peer reviewed research of any design (qualitative, quantitative, mixed methods), *directly* focused on the phenomenon of interest. | 1) tangentially mentioned percentage of patients viewing test results or mentioned patients hypothetically interested in viewing test results;  2) articles addressing other forms of ICT and patient access to information other than test results;  3) focused on genetic portals and inpatient portals for hospitalized patients;  4) focused on software development and implementation as well as portals developed for research purposes or studies using hypothetical scenarios on simulated patients;  5) studies situated outside of high resource countries;  6) review papers and grey literature. |
